# A case of complicated fungal ulcerative keratitis: a rare clinical image

**DOI:** 10.11604/pamj.2026.53.5.48288

**Published:** 2026-01-07

**Authors:** Switi Jawade, Ranjana Sharma

**Affiliations:** 1 Department of Obstetrics and Gynaecology Nursing, Shalinitai Meghe College of Nursing, Salod (Hirapur), Datta Meghe Institute of Higher Education and Research (DU), Sawangi Meghe Wardha, Wardha, India,; 2 Department of Medical Surgical Nursing, Shalinitai Meghe College of Nursing, Salod (Hirapur), Datta Meghe Institute of Higher Education and Research (DU), Sawangi Meghe Wardha, Wardha, India

**Keywords:** Keratitis, corneal ulcer, amniotic membrane, eye infection

## Image in medicine

Corneal ulcer or ulcerative keratitis is an ophthalmologic emergency or infective condition of the cornea due to permanent altered vision or perforation of the eye. A corneal ulcer is usually associated with tissue damage and necrosis, and vision loss. In the United States, the yearly occurrence of corneal ulcer is estimated at 30,000 to 75,000 cases, with 12.2% of corneal transplants performed to address infectious keratitis. A 62-year-old male patient presented to the ophthalmology department with acute ocular complaints. The patient reported redness, reduced visual acuity, blurred vision, excessive watering discharge, severe burning pain in the eye, photophobia, and persistent foreign body sensation. Symptoms were progressive and significantly affected daily activities. Comprehensive ocular evaluation was undertaken, and external eye examination revealed conjunctival erythema and signs of inflammation. Slit-lamp examination shows a corneal epithelial defect with stromal thinning, suggestive of an active corneal ulcer. Confocal microscopy was performed, which identified features consistent with fungal involvement of the cornea. Based on clinical findings and investigation, a final diagnosis is fungal corneal ulcer (fungal ulcerative keratitis). Differential diagnosis considered included bacterial keratitis, herpes simplex virus keratitis, and acanthamoeba keratitis, which were excluded on clinical and investigative findings. The patient received prompt medical management and subsequently underwent amniotic membrane transplantation. At the time of reporting, the patient had been referred to a specialized unit; short and medium-term plans were not available at the time of this report.

**Figure 1:**
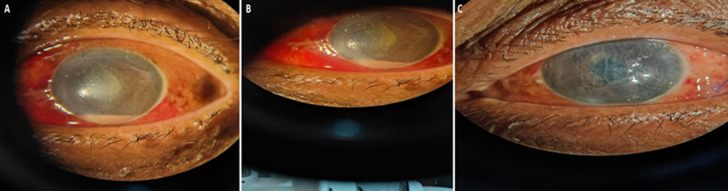
A) whitish or greyish opacity of the cornea; B) cloudy cornea; C) corneal ulcer

